# Tezepelumab improved chronic eosinophilic pneumonia in severe asthma patients with liver cirrhosis

**DOI:** 10.3389/fmed.2024.1381261

**Published:** 2024-06-11

**Authors:** Mizuki Inaba, Yasuo Shimizu, Yusuke Nakamura, Hiroaki Okutomi, Akihiro Takemasa, Seiji Niho

**Affiliations:** ^1^Department of Pulmonary Medicine and Clinical Immunology, Dokkyo Medical University, Mibu, Tochigi, Japan; ^2^Respiratory Endoscopy Center, Dokkyo Medical University Hospital, Mibu, Japan

**Keywords:** eosinophilic pneumonia, asthma, tezepelumab, thymic stromal lymphopoietin, liver fibrosis, sinusitis, eosinophilia, oral corticosteroids

## Abstract

Systemic administration of corticosteroids is used in the treatment of chronic eosinophilic pneumonia (CEP). However, in patients with CEP as well as other comorbidities, the adverse effects of corticosteroids should be minimized as much as possible. A 71-year-old woman was presented with aggravating asthma with CEP and sinusitis, and she had uncompensated liver cirrhosis (LC) with a Child-Pugh score of 7. Initial treatment with a low dose of oral corticosteroids (OCSs) in combination with tezepelumab, an anti-thymic stromal lymphopoietin (TSLP) antibody, resulted in rapid improvement of asthma and CEP without deteriorating LC. Sinusitis also improved after ceasing OCS. This case suggested that tezepelumab may be useful as a treatment option for patients with CEP, especially those with liver dysfunction.

## 1 Introduction

Asthma and chronic eosinophilic pneumonia (CEP) can be comorbid, and studies have shown that oral corticosteroids (OCSs) are successful in treating CEP (1). The metabolism of corticosteroids (CSs) in the liver is impaired in patients with severe liver cirrhosis (LC), and CS may further aggravate liver function and increase the risk of impaired consciousness by elevating the amount of circulating NH_3_ (2). Therefore, providing treatment without worsening liver function is a challenge for patients with LC. Herein, a patient presented with shortness of breath and progressive hypoxemia, which was caused by poorly controlled asthma with CEP, complicated by sinusitis and LC. Initial treatment with a low dose of OCS in combination with tezepelumab, an anti-thymic stromal lymphopoietin (TSLP) antibody, resulted in rapid improvement of asthma and CEP without deteriorating LC. Furthermore, sinusitis also improved after ceasing OCS. This is the first case report of a successful CEP treatment with tezepelumab. Considering that there was a report of successful biologic therapy without OCS for CEP (3), when CEP patients have LC, the initial induction may be conducted with biologics alone, and OCS can be added after assessing the response to biologics.

## 2 Case presentation

**Figure 1:**
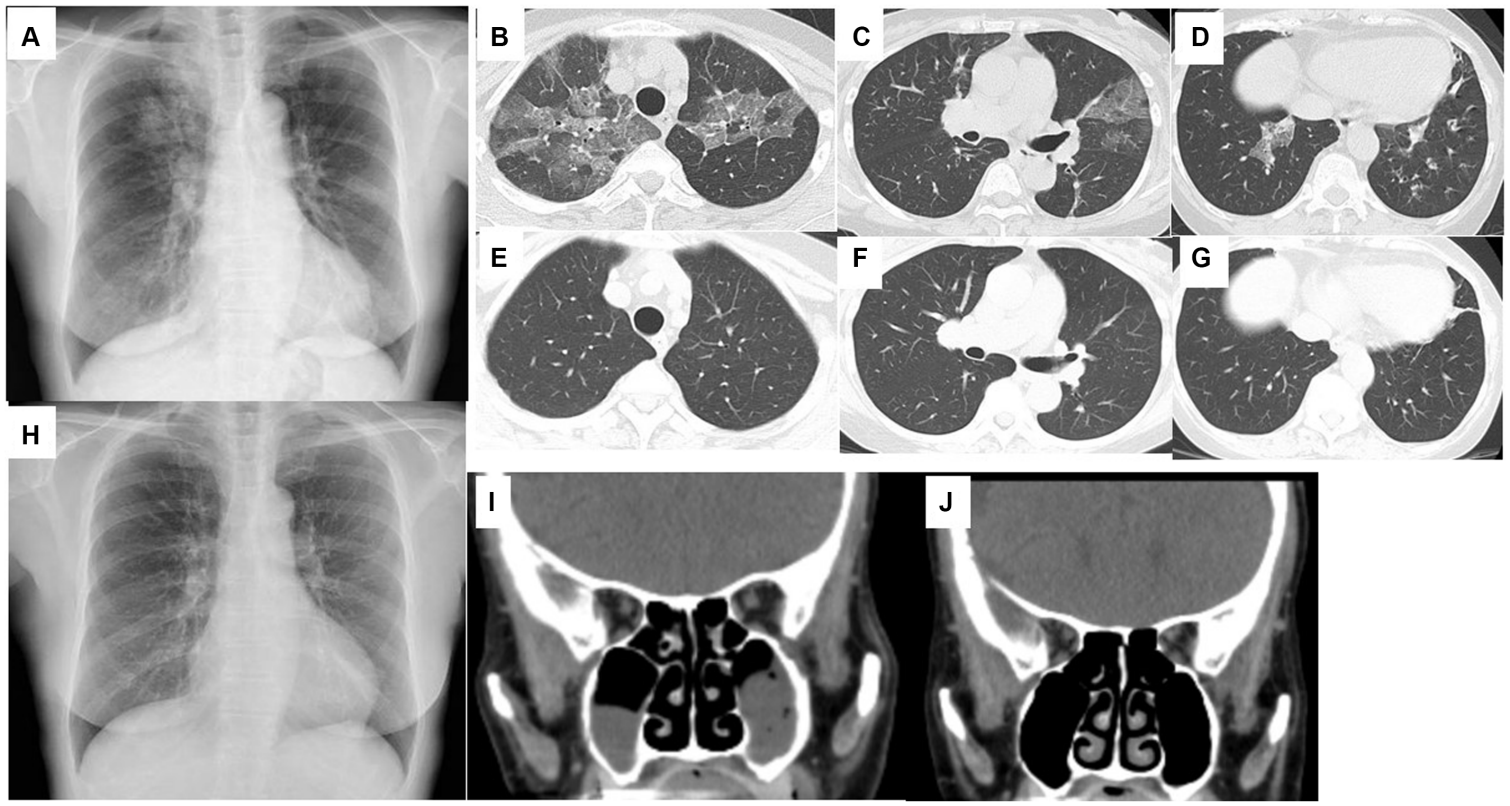
Chest X-ray **(A)** and chest CT **(B–D)** on the day of the first visit to the hospital. Bilateral ground-glass opacities in the upper lobes and bronchial wall thickening in the lower lobes. Chest X-ray on day 10 from starting therapy **(H)**. Bilateral ground-glass opacities disappeared. Chest CT performed 1 month after starting therapy **(E–G)**. Bilateral ground-glass opacities and bronchial wall thickening disappeared. Sinus CT on the day of the first visit to the hospital **(I)**. Bilateral sinusitis exists in the maxillary sinus. Sinus CT 3 months after starting therapy **(J)**. Bilateral sinusitis was improved.

**Figure 2:**
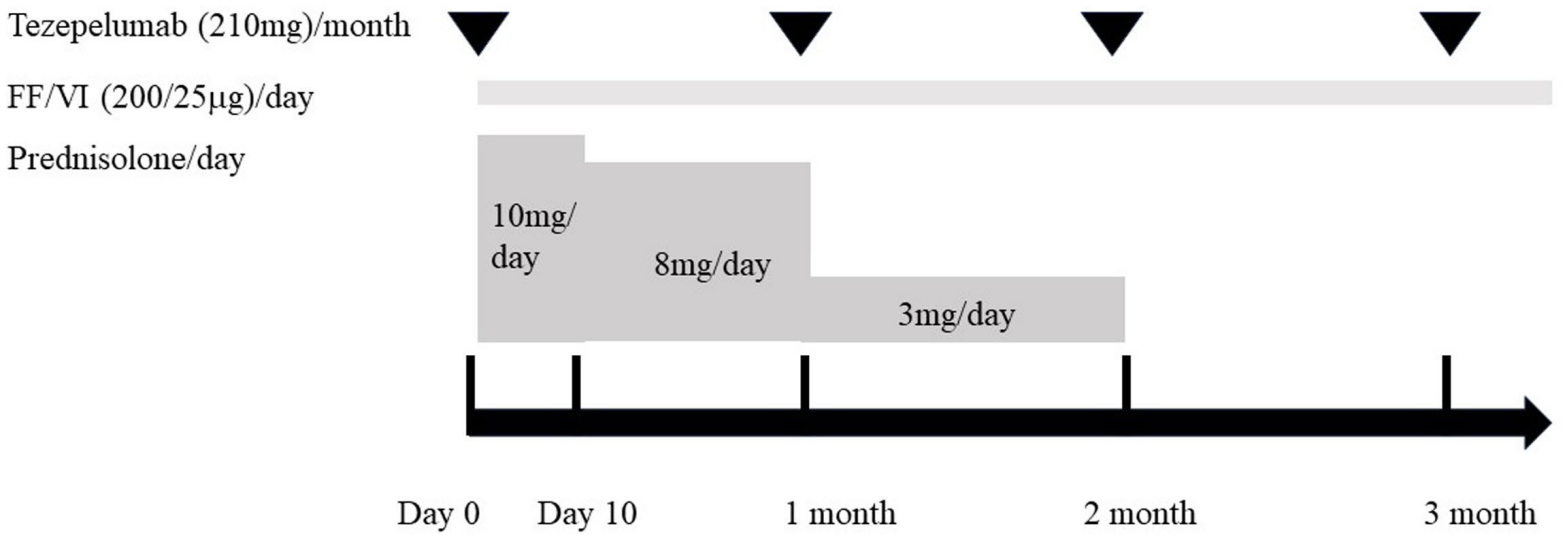
Clinical course of therapy for the present case. Initial therapy was started (day 0) with the combination of oral corticosteroid (prednisolone, 10 mg/day), inhaled fluticasone furoate/vilanterol (FF/VI, 200/25 μg/day), and tezepelumab (210 mg/month). Prednisolone was administered at 10 mg/day for 10 days. Then, the dose was reduced to 8 mg/day and continued for 1 month before being reduced to 3 mg/day during the next month. Two months after starting treatment, prednisolone was discontinued, while FF/VI and tezepelumab were continued.

**Figure 3:**
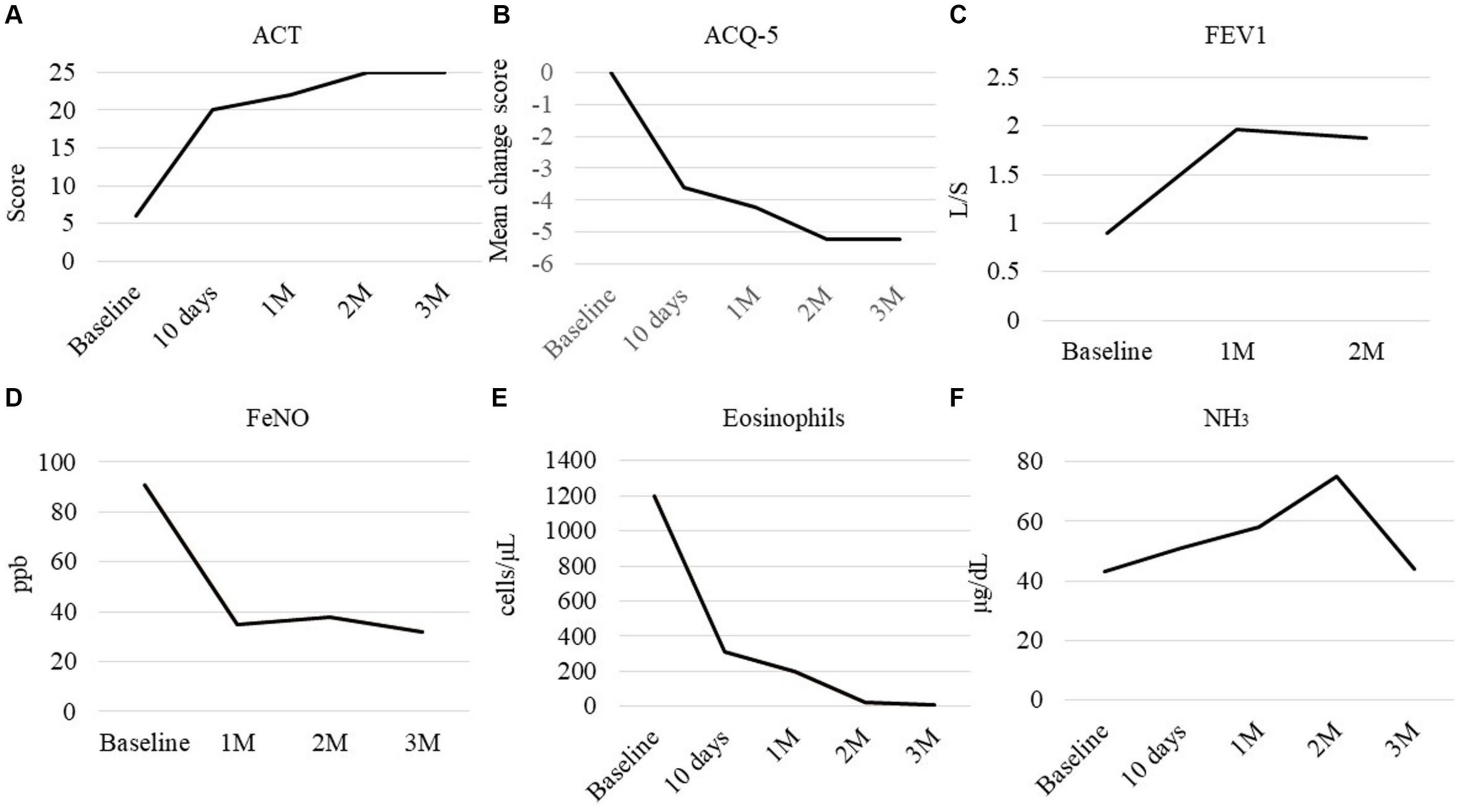
Clinical course of the parameters. **(A)** Asthma control test (ACT), **(B)** mean change from the baseline score of the asthma control questionnaire (ACQ-5), **(C)** forced expiratory volume in 1 s, **(D)** fractional exhaled nitric oxide (FeNO), **(E)** peripheral eosinophil count, and **(F)** NH_3_ in peripheral blood. Baseline: measured on day 0, before starting therapy.

A 71-year-old woman was presented in our hospital with a 2-month history of productive cough, shortness of breath, and hypoxia to SpO_2_ of 92% in room air at a KT of 36.8°C. Auscultation revealed wheezing in both lungs. The respiratory symptoms were severe, presenting an asthma control test score of 6 points and a mean asthma control questionnaire score of 5.2 points. Laboratory examination revealed a normal leukocyte concentration of 5,800 cells/μL but an eosinophilia content of 1,200 cells/μL (20.7%) and reduced platelets (9.3 cells/μL), as well as 66% prothrombin activity and 3.3 g/dL of albumin. Liver enzymes were found in high concentrations, with 3.05 mg/dL of total bilirubin, 1.11 mg/dL of direct bilirubin, 1.94 mg/dL of indirect bilirubin, 158 U/L of alkaline phosphatase, and 43 μg/dL of NH_3_. Eosinophils in sputum were prominent, exhibiting an average of 10–20 cells/field of view, measured by optical microscopy at 200× magnification on five fields. Chest X-rays showed infiltration shadows in the right upper and lower lung fields, chest computed tomography (CT) revealed predominant bilateral infiltration shadows in the upper lobes, which extended to both lower lobes, and sinus CT showed bilateral sinusitis (Figures 1A–D,I). Spirometry indicated a severe obstruction with a forced expiratory volume in 1 s (FEV_1_) of 0.90 L/s and %FEV1 of 50.8%, and the fractional exhaled nitric oxide (FeNO) content was 91 ppb. The clinical diagnosis was CEP complicated by asthma, based on asthma-like symptoms, typical CEP shadows in the lungs, increased peripheral blood eosinophils, and prominent sputum eosinophils, as well as physical examination that revealed no collagen vascular disease (CVD) or rheumatoid arthritis. The patient had been sober for several years, but considering the uncompensated LC and a Child-Pugh score of 7, bronchoscopy was not performed because of the risk of coma following anesthesia (4). Treatment began with prednisolone (10 mg/day), inhaled fluticasone furoate/vilanterol (FF/VI, 200/25 μg/day), and tezepelumab (210 mg/month) (Figure 2). After 10 days, the asthma symptoms and chest XP markedly improved (Figure 1H), and after 1 month, the bilateral shadows disappeared (Figures 1E–G). Based on these improvements, the OCS dose was reduced to 3 mg/day. After 2 months of therapy, the asthma symptoms, pulmonary function, circulating eosinophils, and FeNO content markedly improved, but the NH_3_ content increased from 43 μg/dL before therapy to 75 μg/dL at 2 months (Figures 3A–F). The blood tests performed on the day of the first visit provided no findings suggestive of CVD. Thus, OCS was terminated at 2 months and FF/VI and tezepelumab were continued. One month after the cessation of OCS, NH_3_ was reduced to a baseline level of 43 μg/dL, and the other parameters and asthma symptoms remained under control, with no recurrence of CEP. At the same time, a marked improvement in sinusitis was observed (Figure 1J).

## 3 Discussion

In the treatment of CEP, the recommended initial OCS dose is 0.5 mg/kg, and because the patient weighed 49.4 kg, 25 mg/day would usually be used (1). However, this case was complicated by uncompensated LC. In LC, CS metabolism is impaired, and OCS administration does more harm than good, worsening the liver function and increasing the risk of coma, infection, diabetes, and gastrointestinal bleeding due to varices from the esophagus to the stomach. Therefore, the starting dose of OCS was reduced, and biologics were used in combination. Previous reports of long-term safety with tezepelumab were limited to non-asthmatic pulmonary eosinophilia or high OCS user patients in some of the trials (5). The treatment of the patients in the study showed no effects on liver function and no change in liver enzymes (6). As a result, it is suggested that tezepelumab has a low risk of liver injury; therefore, tezepelumab was used herein.

In the future practice of this patient, careful attention should be given to the development of eosinophilic granulomatosis with polyangiitis (EGPA). This patient had nasal polyps and elevated eosinophils, but the myeloperoxidase–anti-neutrophil cytoplasmic antibody (MPO-ANCA) was negative. However, since ANCA-negative EGPA patients also exist (7), careful attention to the development of EGPA is warranted. In the present patient, currently, 5 months have passed since OCS cessation, but no development of EGPA has occurred under tezepelumab therapy.

In conclusion, tezepelumab may be a treatment option for CEP and have a rapid sparing effect on OCS, safely leading to a reduced risk of OCS, even in LC patients. Moreover, TSLP activates group 2 innate lymphoid cells in the liver, promoting liver fibrosis (8, 9). Blocking the function of TSLP may help mitigate the worsening of LC.

## Data availability statement

The original contributions presented in the study are included in the article/supplementary material, further inquiries can be directed to the corresponding author.

## Ethics statement

Written informed consent was obtained from the individual(s) for the publication of any potentially identifiable images or data included in this article.

## Author contributions

MI: Conceptualization, Data curation, Formal analysis, Investigation, Project administration, Visualization, Writing – original draft, Writing – review & editing. YS: Conceptualization, Data curation, Investigation, Project administration, Visualization, Writing – original draft, Writing – review & editing. YN: Writing – original draft, Writing – review & editing. HO: Writing – original draft, Writing – review & editing. AT: Writing – original draft, Writing – review & editing. SN: Writing – original draft, Writing – review & editing.
